# Countries vindicate cautious stance on e-cigarettes

**DOI:** 10.2471/BLT.14.031214

**Published:** 2014-12-01

## Abstract

Countries signed up to an international tobacco control treaty have embraced controversial WHO proposals to regulate electronic cigarettes. Armando Peruga tells Fiona Fleck why.

Q: What are the health risks of using e-cigarettes and how do they differ from conventional cigarettes?

A: Conventional cigarettes burn the tobacco leaf. E-cigarettes – the most common type of what we call electronic nicotine delivery systems or ENDS – vapourize liquid that contains nicotine for the purposes of inhaling. These devices have become popular over the last four or five years, so there are only a few studies on the health risks and we don’t know the long-term effects. We don’t have epidemiological studies, such as those on the links between tobacco and cancer, because such studies take decades to complete. We do, however, have some evidence and part of the debate is about how to draw conclusions from this. The WHO report released at the end of August took into consideration contributions of more than a 100 scientists and regulators, and covers three areas where we believe there is enough evidence to draw conclusions: the health risks of nicotine, of inhaling other toxic emissions contained in these products and of inhaling second-hand emissions.

Q: Why? The harmful parts of cigarettes are surely the tar and various toxic additives.

A: Nicotine is highly addictive and we don’t want non-smokers to start using it. It has a role in neuro-degeneration and there is evidence of brain development problems in children and fetuses that have been exposed to nicotine. WHO does not recommend the use of any form of nicotine for those who have never smoked or for children and pregnant women, and our report on e-cigarettes is consistent with this position.

Q: At a meeting in Moscow in October of the countries signed up to the WHO Framework Convention on Tobacco Control (FCTC), the countries welcomed WHO’s proposals for regulating e-cigarettes and other similar devices. Has WHO’s cautious stance on these products been vindicated?

A: The countries agreed on a set of goals in line with the WHO recommendations. These seek to prevent non-smokers and young people ever starting to use ENDS; to protect bystanders from ENDS’ emissions; to minimize the potential health risks posed by ENDS and to challenge unproven health claims used to market these products. To achieve their goals, the countries said that they would deploy any regulatory measures they see fit to limit the use of ENDS or ban their use altogether.

Q: Critics of WHO’s cautious stance on e-cigarettes argue that these devices are important for helping people quit smoking in the same way that people with heroin dependence are treated with methadone. Do you feel that this is a realistic comparison and can e-cigarettes be used effectively to help people stop smoking?

A: For now the evidence is inconclusive about whether ENDS can be an effective smoking cessation aid. The debate about ENDS will indeed continue. As pointed out by the WHO report to the Conference of Parties, the evidence and recommendations presented in it are subject to rapid change and in coming years we will build up a solid body of evidence on this matter that will allow us to reach a definitive conclusion.

Q: So shouldn’t people use nicotine delivery products to help them give up smoking?

A: WHO recommends licensed forms of nicotine replacement therapy to help adult smokers quit. While the nicotine in electronic cigarettes does not pose additional health risks for adult smokers, as our report notes, the evidence for the effectiveness of e-cigarettes as a method for quitting smoking is limited and requires further research.

Q: What is the problem with second-hand fumes from e-cigarettes?

A: Currently there are no studies that link the exhaled aerosol from e-cigarettes to specific diseases, but we know that it contains nicotine and particulate matter. These are the tiny particles to which some toxicants are attached. We also know that when this aerosol is exhaled into the indoor air, the background level of these particulates and of nicotine goes up. WHO has long maintained that there is no safe level of such particulates and that we should minimize these levels as far as possible, regardless of their source. The level of particulate matter from e-cigarettes is lower than from conventional cigarettes, but there is a dose–response relationship, which means that the higher the concentration – the greater the health risks. So why expose people unnecessarily to a level of particulate matter that is higher than the background levels?

Q: What toxic substances are produced by vaping?

A: There are about 500 e-cigarette brands out there, and only a few have been analysed. This literature shows a great variety in the levels of the toxicants and nicotine they produce. Some of the analysed brands are known to have very low toxic emissions compared with conventional cigarettes. Some e-cigarettes have few and low levels of toxicants, but some contain levels of cancer-causing agents, such as formaldehyde, that are as high as those in some conventional cigarettes. Nevertheless, average e-cigarettes are likely to be less toxic than conventional cigarettes, although they are not without risks. Some people say these risks are very very low, but our question is “how low?” If smoking a cigarette is like jumping from the 100th floor, using an e-cigarette is certainly like jumping from a lower floor, but which floor? We don’t know.

“Some e-cigarettes have few and low levels of toxicants, but some contain levels of cancer-causing agents, such as formaldehyde, that are as high as those in some conventional cigarettes.”

Q: When are we likely to know more?

A: Some research programmes are already under way but given that e-cigarettes have been popular in the last four or five years, research has barely started and it’s early days yet. It would take about five or 10 years before we have evidence that could change the current picture. Regulation can help generate more evidence by requiring manufacturers to disclose the ingredients in these products and by requiring surveillance to be carried out on their effect on human health.

Q: The European Union (EU) is concerned about refillable e-cigarettes and some EU countries want to ban them. Why are they considered particularly harmful for human health?

A: There are several reasons. First, the users fill the e-cigarette’s container themselves, so it’s not the manufacturer who establishes the level of nicotine, but the user. The liquid may accidentally come into contact with the users’ skin and give them nicotine poisoning and children might drink it. The United States of America, the United Kingdom of Great Britain and Northern Ireland and possibly other countries are surveying such poisoning accidents. Both countries have seen a tremendous increase in nicotine poisoning reports, often involving children. Another concern about refillable devices is that they can be used for illegal substances. So there are many safety issues concerning refillable e-cigarettes that can be minimized with the appropriate regulation.

Q: Why then has there been a controversy over WHO’s proposals to regulate e-cigarettes in the media and on social media?

A: WHO received several letters from health professionals and scientists, including one from a group of 53 people and another from 133 people. The first was from people in the United Kingdom, the United States and some other developed countries. The second was from people living all over the world. These letters gave different interpretations of the scientific evidence and while the second concluded that significant regulatory action was needed, the first did not consider such regulation justified. When compiling our report, we gave due consideration to their arguments and weighed our assessment of the evidence against theirs, reflecting on what was reasonable on both sides.

Q: So are they right or wrong?

A: There is some difference of opinion on how to interpret the science. But the main disagreement is over what to do in terms of regulation and what regulation can achieve, given that current scientific evidence on e-cigarettes is limited. Our view is that regulation should bring the best out of any product while minimizing the worst: a very difficult balance to achieve. In this case, probably, there is no clear right or wrong. That’s why we have to be very careful in our report about limiting our own conclusions to a small number of regulatory areas. While we think that there is enough evidence to regulate some key aspects of e-cigarettes, ultimately, the proof of the pudding is in the eating. The effect of such regulations also needs to be monitored closely. In the end, the key issue is whether e-cigarettes can truly and safely move people out of conventional cigarettes into other less risky forms of nicotine and, eventually, out of nicotine altogether. There is very little science to assess if they can do this, but a lot of debate.

“The key issue is whether e-cigarettes can truly and safely move people out of conventional cigarettes into other less risky forms of nicotine and, eventually, out of nicotine altogether.”

Q: What role do multinational companies play in the new and growing e-cigarettes market?

A: Some people say that WHO is more concerned about beating the tobacco industry than ending the smoking epidemic, even if one of their products, namely e-cigarettes, can help. This view is naive, because the tobacco industry only markets products that it considers capable of perpetuating their core and most profitable product – conventional cigarettes. Unlike those who advocate e-cigarettes as a form of harm reduction, the tobacco industry is clearly not convinced that e-cigarettes will advance smoking cessation efforts, otherwise they would not be aggressively buying up small e-cigarette companies. There have been an estimated 100 million deaths due to tobacco over the last century. How can we trust the companies that caused this human disaster? By appearing to offer a solution with one hand, while continuing to create mass destruction with the other, the tobacco industry is trying to regain the respectability it lost long ago. The manufacturers of cigarettes and other tobacco products cannot be legitimate partners in any public health discussion.

Q: By burdening small e-cigarette companies with regulation, won’t you be giving a free hand to multinational tobacco companies which are keen to take over the e-cigarettes market?

A: The tobacco industry is already overpowering the smaller e-cigarette manufacturers as a result of market competition and in the absence of significant regulation. The fate of the small e-cigarette producers is being decided by the tobacco industry, which is attacking other players with the intention of expelling them from the marketplace so that it can take complete control. E-cigarette regulations are designed to protect public health and to be effective. They should be applied to all market players, large or small, the same way other products are regulated. As indicated in the WHO report, our proposed regulatory measures aim to maximize the potential benefits of e-cigarettes and minimize their risks.

